# Covalent decoration of adenovirus vector capsids with the carbohydrate epitope αGal does not improve vector immunogenicity, but allows to study the *in vivo* fate of adenovirus immunocomplexes

**DOI:** 10.1371/journal.pone.0176852

**Published:** 2017-05-04

**Authors:** Ramona F. Kratzer, Sigrid Espenlaub, Andrea Hoffmeister, Matthias W. Kron, Florian Kreppel

**Affiliations:** 1 Department of Gene Therapy, Ulm University, Ulm, Germany; 2 Chair of Biochemistry and Molecular Medicine, Witten/Herdecke University, Faculty of Health/School of Medicine, Center for Biomedical Education and Research (ZBAF), Witten, Germany; Swedish Neuroscience Institute, UNITED STATES

## Abstract

Adenovirus-based vectors are promising tools for genetic vaccination. However, several obstacles have to be overcome prior to a routine clinical application of adenovirus-based vectors as efficacious vectored vaccines. The linear trisaccharide epitope αGal (alpha-Gal) with the carbohydrate sequence galactose-α-1,3-galactosyl-β-1,4-N-acetylglucosamine has been described as a potent adjuvant for recombinant or attenuated vaccines. Humans and α-1,3-galactosyltransferase knockout mice do not express this epitope. Upon exposure of α-1,3-galactosyltransferase-deficient organisms to αGal in the environment, large amounts of circulating anti-Gal antibodies are produced consistently. Immunocomplexes formed between recombinant αGal-decorated vaccines and anti-Gal antibodies exhibit superior immunogenicity. We studied the effects of the trisaccharide epitope on CD8 T cell responses that are directed specifically to vector-encoded transgenic antigens. For that, covalently αGal-decorated adenovirus vectors were delivered to anti-Gal α-1,3-galactosyltransferase knockout mice. We generated replication-defective, *E1*-deleted adenovirus type 5 vectors that were decorated with αGal at the hexon hypervariable regions 1 or 5, at fiber knob, or at penton base. Surprisingly, none of the adenovirus immunocomplexes being formed from αGal-decorated adenovirus vectors and anti-Gal immunoglobulins improved the frequencies of CD8 T cell responses against the transgenic antigen ovalbumin. Humoral immunity directed to the adenovirus vector was neither increased. However, our data indicated that decoration of Ad vectors with the αGal epitope is a powerful tool to analyze the fate of adenovirus immunocomplexes *in vivo*.

## Introduction

Vectored genetic vaccines which are based on replication-deficient adenovirus (Ad) are highly potent inducers of cellular and humoral immune responses. [1,2] Local delivery of Ad vectors causes transduction of a plethora of different cell types—somatic cells, as well as professional antigen-presenting dendritic cells. [3,4] Post-transduction *de novo* expression of transgenic antigen is followed by antigen processing, peptide presentation in the context of MHC class I molecules, and efficient activation of CD8 T cell responses. Additionally, Ad vectors also induce strong antigen-specific antibody responses. It had been found a characteristic of viral vectors, including adenoviral vectors, to induce a Th1-type immune response which is characterized by a more pronounced cellular immunity and conserved humoral responses. [5–7] Vector-mediated elicitation of a desirable transgene product-specific immunity goes along with generation of vector-specific CD8 T cells and antibodies. [1] Background gene expression from *E1*-deleted Ad vectors accounts for intrinsic vector immunogenicity which can serve to assist the development of cellular immunity, but also can hamper subsequent immunizations. [8,9] Supplementation with adjuvants is a common technique to allow for vaccine dose reduction, to assist in induction and preservation of immune responses, and to induce improved immune profiles.

Adjuvants can be immunostimulants or carriers. A vast number of substances have been and are currently tested for their adjuvantation potential. Immunocomplexation is a successful principle for recognition, phagocytosis, and elimination of pathogens. Coating of antigens or intact pathogens with immunoglobulins and complement proteins causes phagocytic uptake of immunocomplexes by phagocytes (macrophages, neutrophils) via interaction with their surface Fc and complement receptors (FcγR, CR1). Usually, small amounts of antigen are covered, crosslinked and precipitated with excess antibody and subsequently cleared from the circulation. Antigen capture by phagocytes is followed by processing, MHC-related presentation, and induction of adaptive immunity. Distinct types of immunocomplexes and targeting of phagocyte surface receptors, such as Fc receptors and lectins by glycans, bispecific adaptor molecules and monoclonal antibodies, were observed to be favorable in immune response induction. [10–18]

The carbohydrate moiety αGal (alpha-Gal) refers to a terminal α-1,3-glycosidically linked galactose residue within the linear trisaccharide moiety galactose-α-1,3-galactosyl-β-1,4-N-acetyl-D-glucosamine, which is an abundant post-translational glycosylation product that is expressed ubiquitously on cells of non-primate mammals, prosimians and new world monkeys. [19–21] Originally, the αGal epitope was discovered as the root of hyperacute xenotransplant rejection. [22] This terminal residue is transferred to the side chains of glycoproteins and glycolipids by enzymatic activity of α-1,3-galactosyltransferase (α1,3GT, UDP-galactose:β-D-galactosyl-1,4-N-acetyl-D-glucosaminide-α-1,3-galactosyltransferase).

Humans, other primate mammals (apes, old world monkeys), and α1,3GT knockout (KO) non-primate mammals (mice [19] and pigs [23,24]) do not express αGal as a glycosylation product as they lack a functional copy of this enzyme. α1,3GT KO animals have been generated by targeted disruption of the *Ggta1* gene, and α1,3GT KO mice are the only available mammal small animal model without αGal on cell surfaces. [19,20,23–28] All α1,3GT-deficient organisms do not synthesize oligosaccharides with a terminal αGal epitope, but produce antibodies to αGal (anti-Gal). Humans develop high anti-Gal serum titers, a process that is initiated and maintained by nutrition and gastrointestinal bacterial flora. [29,30] The naturally occurring αGal carbohydrate epitope becomes an artificial immunogenic epitope in α1,3GT KO mice, and exposure to αGal induces high titer seroconversion in α1,3GT KO mice (here denominated as anti-Gal mice) [31,32], which is considered to be well comparable to the human situation. [33]

Immunization of anti-Gal mice with αGal-decorated inactivated influenza virus strain A/Puerto Rico/8/34-H1N1 (PR8) has been reported as a promising method to improve *s*.*c*. immune response induction. [17] The principle of αGal/anti-Gal immunocomplexation has also been described to adjuvant immune responses that are directed to inactivated or recombinant αGal-decorated vaccination antigens, such as HIV gp120 protein [14,15], Ovalbumin liposomes [16], and tumor cells. [18] Importantly, to our knowledge, for genetic vaccines no research has been conducted to evaluate whether αGal/anti-Gal immunocomplexation is a functional adjuvant system to improve immunogenicity of a vectored vaccine.

While successful in animal models, up to date no efficacious genetic vaccines for humans are available. This can also be attributed to the fact that current animal models are typically non-permissive for Ad [34], and vector-induced immunity differs significantly from that in humans. First in-man trials (STEP trial/HVTN 502 and Phambili study/HVTN 503) applying an Ad5-based vaccine (*gag/pol/nef*) unexpectedly failed to prevent HIV infection. [35] Surprisingly, uncircumcised Ad5-seropositive men had an increased risk of HIV acquisition after vaccination with an Ad5-based vector, while male circumcision protected against HIV acquisition. [36] Although post hoc statistical and correlative analyses indicated that the puzzling vaccine failure and increased HIV acquisition were likely not due to Ad5 preimmunity [37], these results strongly suggested that the *in vivo* fate of adenovirus immunocomplexes (formed from Ad vectors and anti-Ad antibodies) has significant biomedical impact on the generation of antigen- and vector-specific immune responses. Region-specific varying seroprevalences of 37% to up to 95% [38–41] illustrate the biomedical importance of anti-Ad5 humoral immunity, as formation of Ad immunocomplexes represents one of the premier limitations to clinical applicability of Ad5-based vaccines.

Given the reported vaccination success with immunocomplex-forming *s*.*c*. αGal-decorated antigens in Ribi adjuvant, an Ad-based vaccine—as such a highly promising vaccine agent—appeared an ideal candidate to validate this principle. In this work, we transferred the concept of αGal-decoration of vaccines to an adenoviral vectored vaccine system. Ad–αGal/anti-Gal immunocomplexation was evaluated in anti-Gal mice using αGal-decorated Ad vectors expressing intracellular ovalbumin antigen (iOVA).

For our model, we generated replication-defective, *E1*-deleted (Δ*E1*) Ad vectors that were decorated with a defined copy number of αGal epitopes at defined capsomer positions. This was done by defined genetic insertion of cysteine residues into exposed sites of the major capsomers hexon (HVR1 D151C [42], HVR5 T273C [43], 720 copies), fiber (LIGGGCGGGID motif [44], 36 copies) or penton (T343C, 60 copies). The cysteine thiol groups were subsequently chemically modified, i.e. αGal-decorated with thiol-functionalized αGal (αGal—EMCS). This method—known as geneti-chemical modification—was developed by our group. [44]

**Fig 1:**
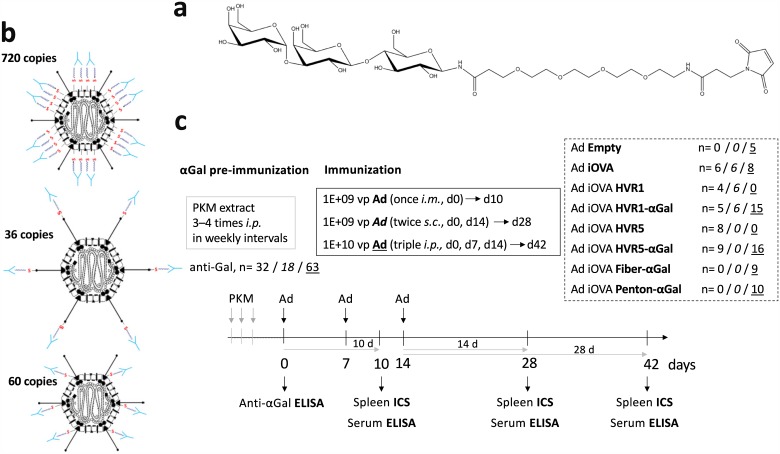
Overview on the anti-Gal system: Concept, components and immunization regimes. (a) Chemical structure of maleimide-activated, thiol-reactive αGal (αGal—EMCS) which was chemically attached to defined surface cysteine residues of Ad vectors. (b) In the anti-Gal αGal epitope-immune mouse model, Ad immunocomplexes (AICs) were formed by delivery of Ad Hexon–αGal, Ad Fiber–αGal or Ad Penton–αGal. (c) αGal preimmunity was established prior to Ad immunization. Immunization doses and routes are indicated. ‘n‘ indicates the number of anti-Gal mice immunized via the *i*.*m*. (n) / *s*.*c*. (*n*) / *i*.*p*. (n) route. Depending on the route, immune responses were analyzed at 10 d, 28 d or 42 d after priming by measuring 1E+05 viable CD8^+^ spleen cells to determine frequencies of IFNγ-producing CD8 T cells.

Delivery of αGal-decorated Ad vectors (Ad–αGal) into anti-Gal mice prompts instantaneous immunocomplexation of Ad–αGal vectors. We re-directed naturally occurring anti-Gal antibodies to defined Ad capsomers. By that we studied the role of immunocomplexation at different Ad capsomer sites in the (i) induction and/or boost of immune responses to vector-encoded transgenic neoantigens, (ii) functionality of αGal/anti-Gal immunocomplexation as an adjuvant system in combination with an Ad-vectored vaccine, as well as (iii) transduction efficiency by Ad immunocomplexes. For an overview on the types of Ad immunocomplexes (AIC) formed in anti-Gal mice by delivery of distinct Ad–αGal vectors, please refer to Fig 1 in the Results section.

For the presented immunological studies, we chose ovalbumin as a well-defined model antigen being used in immunological research during several decades. We generated Ad and Ad–αGal vectors that expressed an intracellular version of OVA (iOVA, OVA_44-285_) as a transgene product. For the transduction studies, we used analogous vectors that expressed enhanced green fluorescent protein (EGFP).

## Material and methods

### Cell lines and cell culture

Cells were passaged twice a week and cultured at 37°C under 5% CO_2_ atmosphere in the appropriate media (MEM, respectively Alpha-MEM) supplemented with 10% fetal calf serum and 1% penicilline/streptomycin/glutamine (Gibco). We used *E1*-complementing N52.E6 cells [45] for Ad production, and A549 cells (#CCL-185, ATCC) for *in vitro* vector characterization.

### Plasmid construction and generation of Ad vectors

All adenovirus vectors (Ad) used in this study were replication-defective *E1*-deleted (Δ*E1*) vectors, based on human adenovirus type 5 (Ad5, PubMed Nucleotide AY339865.1, Δ441–3522) with a genome size of approximately 36 kb. All vectors contained human cytomegalovirus promoter-driven expression cassettes for intracellular chicken ovalbumin (iOVA, OVA_44-285_) for immunological analyses, or enhanced green fluorescent protein (EGFP) for transduction analyses. Cloning details can be obtained from the authors upon request. Ad genomes (*Swa*I-linearized pGS66- or pBacGS66-based infectious plasmids or bacmids) were transfected and amplified in N52.E6 cells, a human amniocyte-derived *E1*-complementing cell line. [45] After amplification, the vectors were purified by a double CsCl density gradient centrifugation. A DNA-based Slot Blot procedure [46] was employed to determine infectious and physical particle titers as described previously. Physical titers were further determined by measurement of optical density of denaturated particles at 260 nm (OD_260_).

### Geneti-chemical modification (glycosylation, αGal-decoration) of Ad vectors

Thiol reactivity-based chemical modification of Ad vectors based on point mutation of the Δ*E*1 Ad genome using Counter Selection BAC Modification Kit (Gene Bridges) according to the manufacturer‘s instructions to introduce cysteine residues at specific capsomer sites. Oligomer sequences can be obtained upon request. Thiol-reactive cysteines were located within hypervariable regions 1 and 5 of hexon (HVR1 D151C [43], HVR5 T273C [42]), next to the RGD loop of penton base (T343C), or within the HI loop of fiber knob (LIGGGCGGCID [44]). All mutations were verified by sequencing.

To obtain thiol-reactive αGal, the reducing terminus of galactose-α1,3-galactosyl-β1,4-glucose (Linear B6 trisaccharide, Dextra Laboratories) was converted to an amine group by Kochetkov amination reaction of 10 mg αGal in aqueous solution (saturated with (NH_4_)_2_CO_3_ and in the presence of additional 200 mg (NH_4_)_2_CO_3_) at room temperature (RT) under stirring for five days. By the second day, another 200 mg (NH_4_)_2_CO_3_ were added. After shock freezing in liquid N_2_, αGal-NH_2_ was lyophilized, dissolved in 1 ml of distilled water, again lyophilized and stored at –80°C in argon atmosphere. 6 mg of reaction product αGal-NH_2_ in 120 μl Dimethyl sulfoxide (DMSO) was reacted with 14.66 mg of heterobifunctional crosslinker Sulfo-EMCS (N-ε-Maleimidocaproyloxy]-sulfosuccinimidylester, Thermo Fisher) in 120 μl water-free DMSO overnight at RT in argon atmosphere (αGal-NH_2_ to Sulfo-EMCS ratio of 1:3). Reaction mixtures were brought to 2 ml with 20 mM HEPES, pH 7.4 and purified with a Superdex Peptide HR 10/300 GL column with an ÄKTApurifier (GE Healthcare) in two runs of each 1 ml. The resulting pure thiol-reactive αGal derivative was stored at –80°C in argon atmosphere until being used for chemical modification.

Thiol-functionalized Ad vectors were recovered from a CsCl step gradient and were mixed with 20- to 30-fold excess of thiol-reactive αGal in argon atmosphere. For reaction calculation, vector concentration was determined by OD_260_. Reaction time was 2 h at RT. Then the sample was purified from unreacted αGal in a continuous CsCl gradient.

αGal-modification of Ad was proven immunochemically by SDS-PAGE and Western Blot or ELISA. We performed SDS-PAGE of 5E+09 vp Ad–αGal and gel transfer to a Nitrocellulose membrane (Amersham Hybond ECL 0.45 μm, GE Healthcare). For ELISA-based detection of αGal-decoration, Ad–αGal was coated O/N at RT (1E+10 vp in 50 μl/well) to a 96 well ELISA plate (Maxisorp, Thermo Fisher) in carbonate buffer (0.2 M Na_2_CO_3_/NaHCO_3_, pH 9.5). Plates were repeatedly washed, and blocked for 2 h at RT with 250 μl of 5% BSA in DPBS containing 1 mM Ca^2+^ and 0.5 mM Mg^2+^ ions. 100 μl of Biotin-labelled BS-Lectin from *Bandeiraea simplicifolia* (100 μg/ml; BS-I Isolectin B4 Biotin Conjugate, Sigma) which recognizes terminal α-D-galactosyl residues was added instead of a primary antibody and incubated for 2 h at RT. Then, 100 μl of HRP-labelled Streptavidin (Streptavidin—Peroxidase from *Streptomyces avidinii*, Sigma) was added and incubated for 1 h at RT. Signal was read out with an ELISA reader (Multiskan Ex, Thermo Scientific) at 491 nm and corrected for background at 620 nm after incubation with o-Phenylenediamine dihydrochloride substrate solution (Life technologies) and reaction stop with 1 M H_2_SO_4_.

### *In vitro* Ad immunocomplexation after anti-Gal IgG purification from human serum

Chromatography columns were packed with Melibiose-Sepharose (Sigma) which binds the anti-Gal IgGs that are contained in liquid *i*.*v*. human normal IgG (Privigen, CSL Behring). The anti-Gal IgG eluate was enriched by Amicon ultrafiltration (Merck). The enriched anti-Gal IgG fraction was incubated with excess Ad in DPBS O/N at RT to deplete residual anti-Ad IgG. To clear anti-Gal IgG from Ad/anti-Ad IgG immunocomplexes, we applied a Superdex Peptide HR 10/300 GL column (GE Healthcare). Western Blot and ELISA verified identity of purified IgGs as anti-Gal IgG, and proved absence of anti-Ad IgGs.

### Animal experiments

α1,3GT knockout mice (anti-Gal mice, C57BL/6N background) were originally obtained from Peter J. Cowan, Victoria, Australia, and bred and maintained at the animal breeding facility at Ulm University, Germany. C57BL/6N and BALB/c control mice were obtained from Charles River animal breeding colony. All strains were kept in specific pathogen-free environment in individually ventilated cages (Tecniplast) and fed with sterilized Ssniff laboratory rodents feed. Experiments were done with animals of both sexes which resulted in comparable data. Mice were used at a minimum age of 4–6 weeks at the time point of pig kidney membrane (PKM) pre-immunization, and a minimum age of 8–10 weeks at the time point of Ad immunization. All animal experiments were authorized by the German Federal Commission of Animal Protection and conducted according to federal and institutional guidelines.

Experiments were performed in αGal-tolerant C57BL/6N or BALB/c mice, and anti-Gal preimmune α1,3GT KO mice (anti-Gal mice). To generate anti-Gal preimmune status in α1,3GT KO animals, we conducted 3–4 *i*.*p*. injections of each 500 ng of pig kidney membrane (PKM) extract which is rich in αGal-decorated glycoproteins and glycolipids that elicit anti-Gal production. anti-Gal IgG titers after pre-immunization were determined by ELISA of serum obtained by tail vein puncture prior to the actual experiment. Ad vectors were administered to animals with anti-Gal IgG titers of ≥1000 applying one of the following immunization regimes (also refer to Fig 1c in the Results section): (i) *i*.*m*.: one dose of 1E+09 vp in 50 μl, (ii) *s*.*c*.: two doses of each 1E+09 vp in 50 μl in biweekly interval, (iii) *i*.*p*.: three doses of each 1E+10 vp in 100 μl in weekly intervals. The indicated doses refer to physical vector particles. Immunological analyses of cellular and humoral responses were performed at 10 to 42 days after prime immunization. For liver transduction analyses after 72 h, 3E+10 vp were administered *i*.*v*. in a total volume of 200 μl. Mice were sacrificed by an overdose of the inhalation anesthetic isoflurane (Forene, Abbott) and subsequent mechanical rupture of the diaphragm. After inspection of the peritoneal cavity and thorax, whole spleens were removed and kept on ice in sterile 3% BSA in DPBS. Blood samples were obtained by heart puncture and sera were frozen at –20°C. For transduction analyses, livers were perfused before organ removal.

### *In vivo* transduction analyses

To analyze transduction capacity of thiol-functionalized or αGal-decorated Ad vectors, EGFP-expressing vectors were administered *i*.*v*., and liver and spleen transduction was analyzed at 72 h. Cryosection microscopy, fluorimetry, and qPCR were performed as described elsewhere. [43]

### Immune response analyses

Spleen single cell suspensions were prepared as described previously. [47] Restimulation was done for 4 h (37°C, 5% CO_2_) in the presence of 3.3 μg/ml H2^b^-restricted peptides being derived from the transgenic antigen (SIINFEKL, OVA_257-264_) or the Ad vector (FALSNAEDL, DBP_418-426_, Ad5 DNA binding protein) and 6.6 μg/ml Brefeldin A (Sigma) as a protein secretion inhibitor. Whenever we conducted restimulation of spleen cells to detect CD8 T cell responses, we conducted control incubations to exclude unspecific restimulation. For that, we selected one animal per experimental group (subjected to the same immunization protocol, but differing in the administered vector) and incubated spleen cells with an irrelevant peptide in parallel to the restimulations that were done with OVA_257-264_ or DBP_418-426_. Cells were harvested, Fc-blocked for 15 min at 4°C, surface stained with anti-CD8/PacificBlue (clone 53–6.7, BD Bioscience) for 20 min at 4°C in a volume of 50 μl, fixed for 20 min at RT (2% PFA in DPBS), and permeabilized for 15 min at RT (0.5% BSA, 0.5% saponin, 0.05% sodium azide in DPBS). Intracellular cytokine staining (ICS) was done for 30 min at RT in permeabilization buffer containing a mix of three anti-cytokine antibodies, anti-IFNγ/FITC (clone XMG1.2, BD Bioscience), anti-TNFα/APC (clone MP6-XT22, BD Bioscience) and anti-IL2/PE (clone JES6-5H4, BD Bioscience). Frequencies of cytokine-positive, antigen- or vector-specific CD8 T cells after peptide restimulation were measured by flow cytometry using a Beckman Coulter Gallios Flow Cytometer. We measured 1E+05 viable CD8^+^ spleen cells to quantify the percentage of cytokine-expressing cells and mean fluorescence intensity (MFI) of total cell count.

Anti-Ad, anti-OVA and anti-Gal serum IgG titers were determined by ELISA. Heat-inactivated Ad particles (10 min, 60°C; 1.1E+10 vp/ml), OVA or BSA–αGal (Galα1-3Galβ1-4GlcNAc-BSA, 3 atom spacer, Dextra Laboratories; 4 μg/ml) were coated O/N at 4°C in carbonate buffer (0.2 M Na_2_CO_3_/NaHCO_3_, pH 9.5) to Maxisorp plates. Sera were serially diluted in blocking buffer (3% BSA in DPBS) and incubated for 2 h at RT. Secondary antibody (rabbit anti-mouse IgG-HRP, Sigma) was incubated for 1 h at 37°C. Signal was read out with an ELISA reader (Multiskan Ex, Thermo Scientific) at 491 nm and corrected for background at 620 nm after incubation with o-Phenylenediamine dihydrochloride (Life technologies) substrate solution and stopping with 1 M H_2_SO_4_.

### Statistics

Statistical significance was tested by two-tailed unpaired student’s *t*-test assuming equal variances. Only values of P≤0.05 were considered significant and included in the figures.

## Results

### Ad5-based vectors were successfully decorated with the carbohydrate epitope αGal

**Fig 2:**
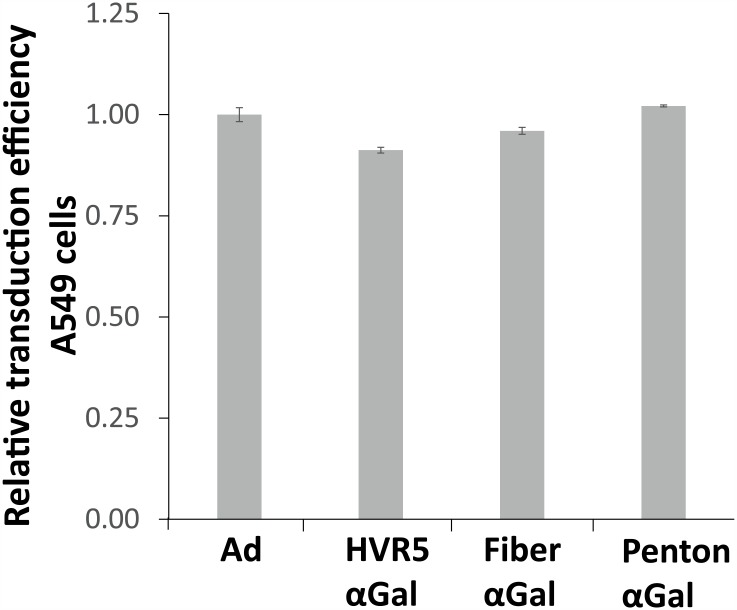
αGal-decoration did not alter *in vitro* transduction capacity of Ad–αGal vectors. For a comparison of transduction capacity, A549 cells were transduced with 500 MOI of EGFP-expressing vectors and analyzed by flow cytometry at 48 h. ‘Ad’ is unmodified control vector Ad EGFP, while the other vectors were αGal-decorated at the indicated capsomer site. The figure shows relative transduction efficiency normalized to Ad control.

To enable covalent surface decoration of antigen-expressing Ad vectors with the trisaccharide αGal, thiol-reactive cysteines were inserted at distinct capsomer sites. αGal was first aminated at its reducing end according to Kochetkov, and the amination was confirmed by ^1^H-NMR (data not shown). Subsequently, aminated αGal was reacted in DMSO with the heterobifunctional crosslinker EMCS to introduce thiol reactivity. The modified product was purified on a Superdex Peptide 10/300 GL column. The purified maleimide-activated αGal epitope (Fig 1a) was reacted with different thiol-bearing Ad vectors (Fig 1b). The percentage of αGal-decoration was determined by an αGal-specific ELISA using BS lectin (*Bandeiraea simplicifolia* BS-I Isolectin B4 Biotin Conjugate) and found to be 85–95%, i.e. 85–95% of the genetically introduced cysteine residues per vector particle were covalently decorated with the αGal epitope. Importantly, the ability of the vectors to transduce A549 cells *in vitro* was not affected by decoration with αGal (Fig 2). This was determined by flow cytometry of A549 cells at 48 h after transduction with EGFP-expressing vectors. Physical and infectious vector particle titers and ratios were determined by a DNA-based Slot blot procedure (Table 1). Physical-to-infectious titer ratios were below 30 for all vectors. This reflects the normal state of inverse bioactivity of adenoviral vectors.

**Table 1:** Infectious and physical vector particle titers of Ad and Ad–αGal vectors.

Ad vector preparation	Physical titer [vp/μl]	Infectious titer [vp/μl]	Physical: Infectious vp ratio
Ad **Empty**	5.65E+08	1.66E+08	3.39
**Ad** iOVA (OVA_44-285_)	1.37E+09	3.95E+08	3.48
Ad iOVA **HVR1**	9.53E+08	1.63E+08	5.84
Ad iOVA **HVR1–αGal**	9.43E+08	6.18E+07	15.26
Ad iOVA **HVR5**	7.31E+08	1.17E+08	6.26
Ad iOVA **HVR5–αGal**	3.16E+08	7.40E+07	4.27
Ad iOVA **Fiber**	5.32E+08	6.76E+07	10.09
Ad iOVA **Fiber–αGal**	5.96E+08	1.08E+08	5.52
Ad iOVA **Penton**	3.50E+08	7.05E+07	4.96
Ad iOVA **Penton–αGal**	3.04E+08	1.03E+08	4.08

Infectious and physical vector particle (vp) titers were determined by a DNA-based Slot Blot procedure. The table summarizes vp titers per microliter [vp/μl] and indicates the ratio of physical to infectious vector particles.

### αGal-decoration had capsomer position-specific effects on the induction of transgene product-specific CD8 T cell responses by Ad–αGal

**Fig 3:**
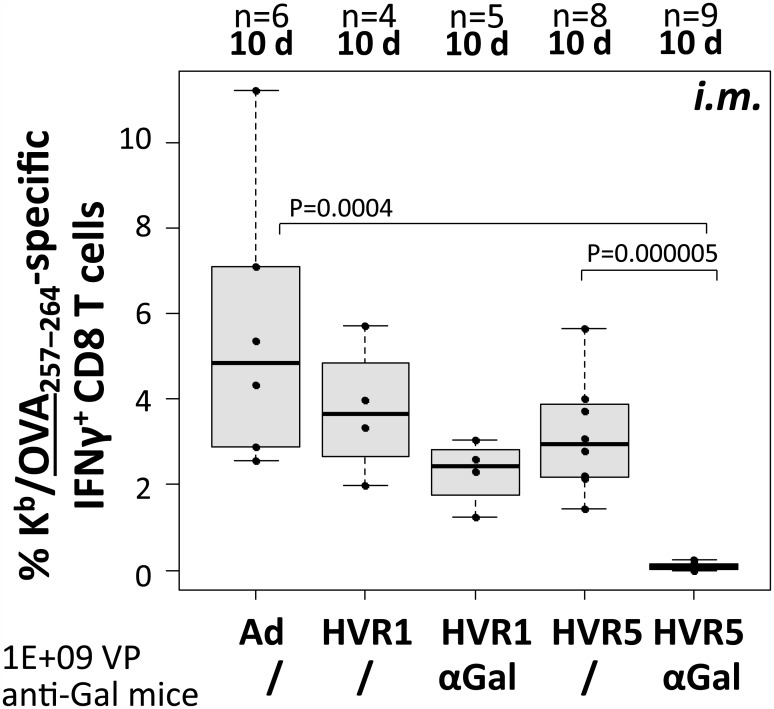
Ad–αGal immunocomplexation after *i*.*m*. immunization significantly decreased antigen-directed CD8 T cell frequencies at day 10. We measured 1E+05 viable CD8^+^ spleen cells to determine antigen (OVA_257–264_)-directed IFNγ-producing CD8 T cell frequencies at 10 d following *i*.*m*. administration of 1E+09 physical vector particles. ‘n’ indicates the number of animals per group. All vectors carried CMV-promoter driven iOVA expression cassettes. ‘Ad’ is unmodified control vector Ad iOVA, while the other vectors were αGal-decorated at the indicated capsomer site in hexon HVR1 or HVR5. P values were calculated by unpaired, two-tailed *t*-test assuming equal variances. Only P values <0.05 are indicated.

We immunized anti-Gal mice (anti-Gal titers ≥1000) with distinct Ad vectors (αGal-decorated vectors vs. undecorated control) according to the regimes depicted in Fig 1c, and then determined antigen-specific CD8 T-cell responses. First, ovalbumin (iOVA)-expressing Ad vectors being decorated at the hexon capsomer (720 copies per particle) were analyzed for their potency to induce OVA-specific CD8 T cell responses. Since it has been reported that immune responses against genetically inserted antigenic peptide epitopes can vary depending on the position at which the peptide epitopes have been introduced into the capsid [48–50], we compared vectors decorated at hexon hypervariable region 1 or 5 (HVR1 or HVR5). Both vectors were decorated to the same extent (90% of cysteines were decorated with αGal). Thus, we could analyze the potential role of αGal’s position at the capsomer hexon, while the same copy number of αGal epitopes was present per particle. Mice were immunized *i*.*m*. with 1E+09 physical vector particles and 10 days later, the frequency of SIINFEKL (OVA_257–264_)-specific CD8 T-cells was determined by *ex vivo* peptide restimulation and intracellular cytokine staining of spleen cells (Fig 3). The data revealed that the introduction of cysteine residues into HVR1 or HVR5 did not impact on the frequency of SIINFEKL-specific CD8 T cells compared to an undecorated control vector (Ad control: 5.6%, HVR1: 3.8%, HVR5: 3.1%). Vector immunocomplexes being decorated with αGal at hexon HVR1 were able to induce SIINFEKL-specific CD8 T cell responses with frequencies comparable to those induced by the undecorated control vectors (HVR1–αGal AICs: 2.7%). However, vector immunocomplexes being decorated with the αGal epitope at HVR5 only induced very low frequencies of SIINFEKL-specific CD8 T-cells (HVR5–αGal AICs: 0.1%, P = 0.0004). These data suggested that the attachment site of αGal at the Ad vector capsid was crucial for the vectors’ ability to induce transgene product-specific CD8 T cell responses. Further, the data indicated that after a single prime immunization, the αGal epitope at HVR1 did not improve transgene product-specific CD8 T cell frequencies.

### αGal-decoration of Ad5 vector capsids did not increase vector-induced transgene product-specific CD8 T cell responses

**Fig 4:**
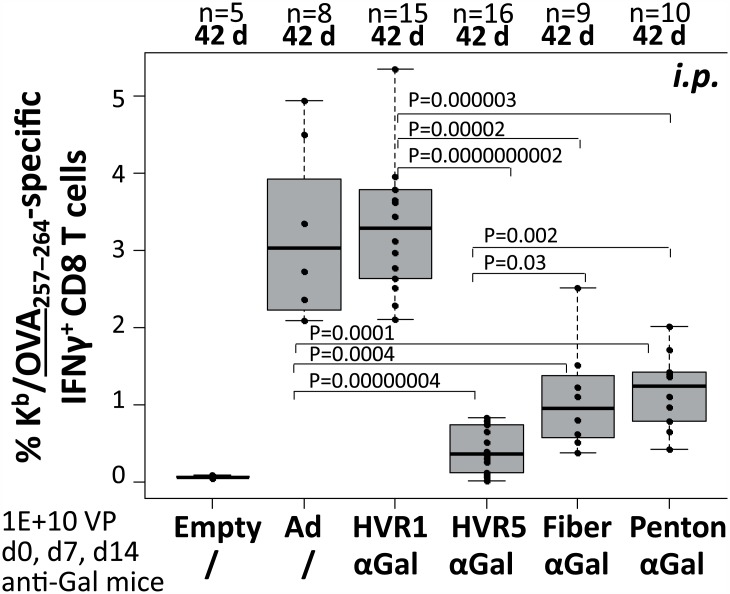
Ad–αGal immunocomplexation following *i*.*p*. homologous prime/boost immunization significantly decreased antigen-directed CD8 T cell frequencies at day 42. We measured 1E+05 viable CD8^+^ spleen cells to determine antigen (OVA_257–264_)-directed IFNγ-producing CD8 T cell frequencies at 42 d after priming, corresponding to 28 days after the third *i*.*p*. vector dose of 1E+10 physical vector particles. ‘n’ indicates the number of animals per group. ‘Empty’ is unmodified control vector Ad Empty which does not carry an expression cassette. All other vectors carried CMV-promoter driven iOVA expression cassettes. ‘Ad’ is unmodified control vector Ad iOVA, while the other vectors were αGal-decorated at the indicated capsomer site in hexon HVR1 or HVR5, fiber or penton. P values were calculated by unpaired, two-tailed *t*-test assuming equal variances. Only P values <0.05 are indicated.

To analyze if the immunization route influenced the induction of transgene product-specific CD8 T cell responses, αGal-decorated vectors were injected *i*.*p*. on days 0, 7, 14, and the frequency of SIINFEKL-specific IFNγ-producing CD8 T cells was determined on day 42 by *ex vivo* peptide restimulation and intracellular cytokine staining of spleen cells (Fig 4). The frequencies of SIINFEKL-specific IFNγ-producing CD8 T cells induced by vector immunocomplexes decorated with αGal at hexon HVR1 were comparable to those induced by the unmodified control vector (Ad control: 3.2%, HVR1–αGal AICs: 3.4%). In contrast, vector immuncomplexes decorated with αGal at hexon HVR5 induced significantly lower frequencies of SIINFEKL-specific CD8 T cells (HVR5–αGal AICs: 0.5%). In addition, vector immunocomplexes decorated with αGal at the fiber knob or the penton base capsomer (36, respectively 60 copies per particle) induced significantly lower frequencies of SIINFEKL-specific CD8 T-cells (Fiber–αGal AICs: 1.1%, Penton–αGal AICs: 1.2%) compared to an unmodified control vector and compared to vector immunocomplexes decorated with αGal at hexon HVR1. Overall, the data indicated that the frequency of transgene product-specific IFNγ-producing CD8 T cell responses induced by Ad5-based vectors could not be increased by decoration of the vector capsid with the carbohydrate epitope αGal. In contrast, depending on the position at which the capsids were decorated, a significant decrease in transgene product-specific CD8 T cell responses was observed.

### αGal-decoration of Ad5 vector capsids did not increase vector-induced transgene product-specific humoral IgG responses or αGal-specific humoral IgG responses

**Fig 5:**
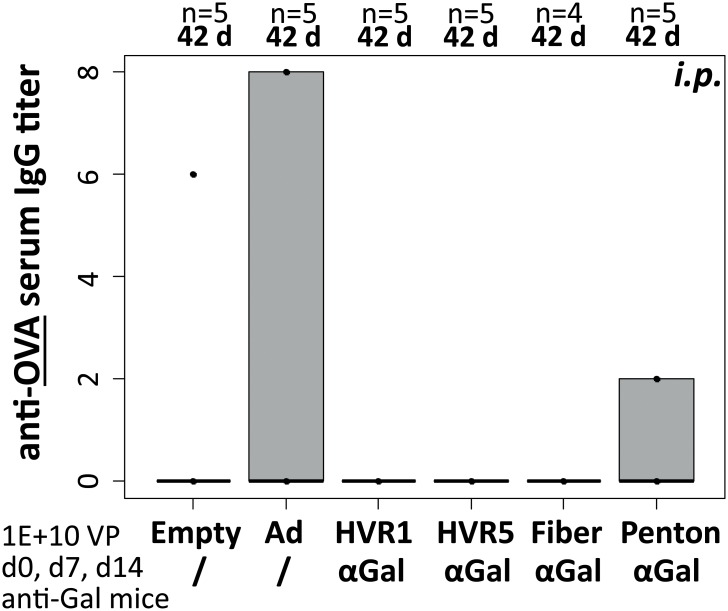
Ad–αGal immunocomplexation following *i*.*p*. homologous prime/boost immunization did not increase induction of antigen-directed humoral response by day 42. Antigen-directed IgG humoral responses (anti-OVA serum IgG titers) were analyzed at 42 d after priming, corresponding to 28 days after the third *i*.*p*. vector dose of 1E+10 physical vector particles. ‘n’ indicates the number of animals per group. ‘Empty’ is unmodified control vector Ad Empty which does not carry an expression cassette. All other vectors carried CMV-promoter driven iOVA expression cassettes. ‘Ad’ is unmodified control vector Ad iOVA, while the other vectors were αGal-decorated at the indicated capsomer site in hexon HVR1 or HVR5, fiber, or penton. P values were calculated by unpaired, two-tailed *t*-test assuming equal variances. Only P values <0.05 are indicated.

**Fig 6:**
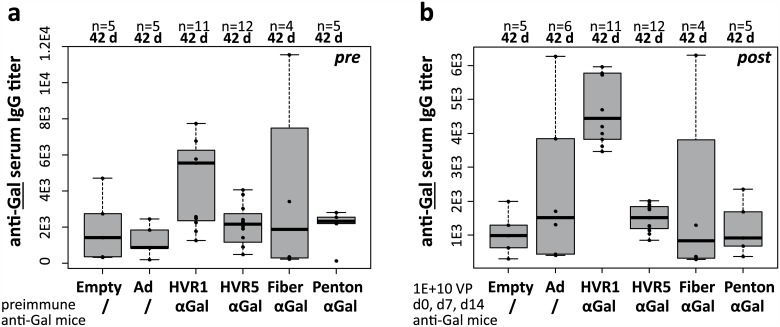
Ad–αGal immunocomplexation following *i*.*p*. homologous prime/boost immunization did not increase anti-Gal-directed humoral response in anti-Gal mice by day 42. αGal epitope-directed IgG humoral responses (anti-Gal serum IgG titers) were analyzed at 42 d after priming, corresponding to 28 days after the third *i*.*p*. vector dose of 1E+10 physical vector particles. (a) Pre-immunization anti-Gal IgG titers as established by 3–4 αGal pre-immunizations with PKM extract were quantified the day before immunization with Ad or Ad–αGal vectors. (b) Post-immunization anti-Gal IgG titers were determined at 28 days after the last *i*.*p*. vector immunization. ‘n’ indicates the number of animals per group. ‘Empty’ is unmodified control vector Ad Empty which does not carry an expression cassette. All other vectors carried CMV-promoter driven iOVA expression cassettes. ‘Ad’ is unmodified control vector Ad iOVA, while the other vectors were αGal-decorated at the indicated capsomer site in hexon HVR1 or HVR5, fiber or penton. P values were calculated by unpaired, two-tailed *t*-test assuming equal variances. Only P values <0.05 are indicated.

In previous immunization experiments applying iOVA-expressing vectors, we found that the intracellular version of the antigen OVA failed to induce humoral anti-OVA IgG responses. To characterize whether αGal-decoration of iOVA-expressing vaccine vectors had an effect on the induction of anti-OVA IgG titers, we analyzed mouse sera by anti-OVA ELISA (Fig 5). We found that the experimental animals did not exhibit anti-OVA IgG titers at 42 d, i.e. 28 days after the third *i*.*p*. immunization with Ad or Ad–αGal immunocomplexes, and hence Ad–αGal immunocomplexes did not increase the vector-induced generation of transgene product-specific IgG responses. A positive control had been included to ensure that measurement of anti-OVA titers had been technically valid. Furthermore, we checked whether immunization of anti-Gal mice with distinct αGal-decorated Ad vectors affected anti-Gal IgG antibody titers (Fig 6). We compared pre-immunization anti-Gal IgG titers after 3–4 pre-immunizations with PKM (Fig 6a) and post-immunization anti-Gal IgG titers of anti-Gal mice at 42 d, i.e. 28 days after the third *i*.*p*. immunization with Ad or Ad–αGal immunocomplexes (Fig 6b). We found that none of the αGal-decorated or undecorated vectors had an impact on anti-Gal serum IgG titers. Comparison of pre- and post-immunization titers did not detect statistical differences. P values of *t*-tests comparing pre- and post-immunization anti-Gal IgG titers in individual experimental groups before and after Ad immunization were in the range of 0.2 to 0.85 (P values not shown).

### αGal-decoration of Ad5 vector capsids did not increase vector-induced vector-specific CD8 T cell responses

**Fig 7:**
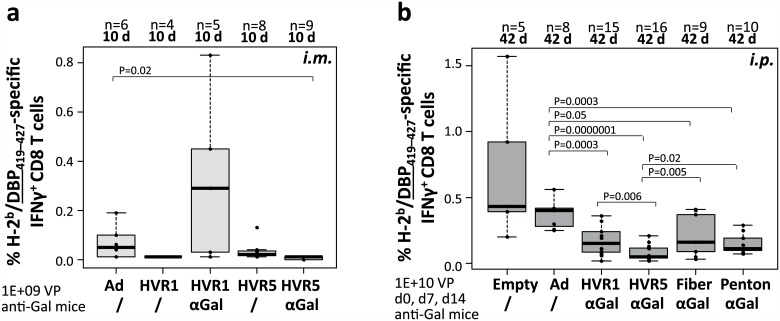
Ad–αGal immunocomplexation following *i*.*m*. or *i*.*p*. immunization of anti-Gal mice significantly decreased Ad vector-directed CD8 T cell frequencies. Mouse samples from Figs 3 and 4 were complementarily analyzed for frequencies of Ad (DBP_419–427_)-directed IFNγ-producing CD8 T cells. (a) Adaptive immune response at 10 d following *i*.*m*. administration of 1E+09 physical vector particles. (b) Adaptive immune response at 42 d following *i*.*p*. administration of three doses of 1E+10 physical vector particles. P values were calculated without outliers by unpaired, two-tailed *t*-test assuming equal variances. Figure only indicates P values <0.05.

An anti-Ad adjuvant effect might be exploited for design of anti-Ad vaccines. To analyze if αGal-decoration affected the induction of Ad-directed CD8 T cell responses, the spleen cells from mice that had been immunized *i*.*m*. (see Fig 3) or *i*.*p*. (see Fig 4) were additionally analyzed by restimulation with an Ad-specific peptide being derived from Ad5 DNA-binding protein (FALSNAEDL, DBP_419–427_). DBP is expressed from the vector, but not packed into the vector particles. Fig 7a depicts frequencies of DBP-specific IFNγ-producing CD8 T cells induced by *i*.*m*. immunization. For vector immunocomplexes decorated with αGal at hexon HVR1, DBP-specific CD8 T cells did not significantly differ compared to those induced by the unmodified control vector (Ad control: 0.1%, HVR1–αGal AICs: 0.3%), while vector immunocomplexes decorated with αGal at hexon HVR5 induced significantly lower frequencies of DBP-specific CD8 T cells (HVR5–αGal AICs: 0.0%). Fig 7b depicts frequencies of DBP-specific IFNγ-producing CD8 T cells induced by *i*.*p*. delivery of vectors. We demonstrated that an Ad with no iOVA expression cassette (Empty) induced comparable frequencies of Ad-directed CD8 T cells as Ad iOVA control vector. For vector immunocomplexes decorated with αGal at hexon HVR1 or HVR5, DBP-specific CD8 T cells were significantly decreased compared to those induced by the unmodified control vector (Ad control: 0.4%, HVR1–αGal AICs: 0.2%, HVR5–αGal AICs: 0.1%). Also vector immunocomplexes decorated with αGal at the fiber or penton base capsomer induced significantly lower frequencies of DBP-specific CD8 T-cells compared to unmodified control vector (Fiber–αGal AICs: 0.2%, Penton–αGal AICs: 0.15%). Overall, Ad vector immunocomplexes decorated with αGal at hexon HVR5 induced significantly lower frequencies of DBP-directed CD8 T cells than all other vectors. Altogether, these data indicated that decoration of Ad5-based vector capsids with the carbohydrate epitope αGal did not increase the frequency of vector-specific IFNγ-producing CD8 T cell responses induced by Ad5-based vectors. In contrast, DBP-specific CD8 T cell responses were significantly decreased.

### αGal-decoration appeared to be a suitable model to analyze *in vivo* transduction by Ad5 immunocomplexes

**Fig 8:**
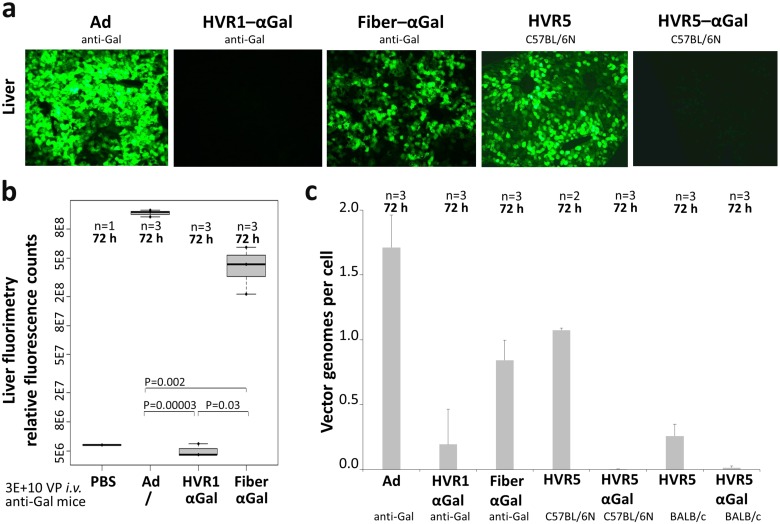
Ad–αGal immunocomplexes altered *in vivo* transduction of liver after systemic delivery of vectors into anti-Gal mice. Transgene product expression was analyzed at 72 h after *i*.*v*. delivery of 3E+10 physical vector particles of EGFP-expressing vectors and differed significantly between vectors decorated with αGal at distinct capsomers. (a) Fluorescence microscopy of liver cryosections depicts transduction results of Ad control vector, HVR1–αGal AICs, and Fiber–αGal AICs in anti-Gal mice, and Ad HVR5 and Ad HVR5–αGal in αGal-expressing C57BL/6N mice. (b) Fluorimetry of anti-Gal mouse livers quantified transgene expression by Ad control vector, or HVR1–αGal or Fiber–αGal AICs. (c) Quantitative PCR detected vector genomes delivered into livers of anti-Gal mice (Ad, HVR1–αGal AICs, Fiber–αGal AICs), and αGal-expressing C57BL/6N mice and BALB/c mice (Ad HVR5, Ad HVR5–αGal). P values were calculated by unpaired, two-tailed *t*-test assuming equal variances.

The previously described experiments showed that decoration of the Ad5-based vector capsid with the carbohydrate αGal did not improve induction of CD8 T cells. However, anti-Gal IgGs present in anti-Gal mice are directed specifically to the decorated capsomer sites in a way that a universal carbohydrate-directed antibody can be exploited as an artificial capsomer-specific antibody. Hence, we used the anti-Gal mouse model to study *in vivo* transduction by defined Ad immunocomplexes. Analogously to iOVA-expressing vectors, we generated a set of vectors which expressed enhanced green fluorescent protein (EGFP) to allow for quantification of transgene product expression. Transgene product expression in liver as the major target organ after *i*.*v*. administration of Ad was analyzed at 72 h after injection of 3E+10 physical vector particles. Cryosection fluorescence microscopy (Fig 8a) showed that unmodified Ad control vector induced strong liver transduction in anti-Gal mice, while the transduction capacity of hexon HVR1–αGal AICs and Fiber–αGal AICs was completely, respectively partially abolished. These results were verified by fluorimetry of liver (Fig 8b). Interestingly, as observed for immune responses, vectors being decorated with αGal at hexon HVR5 showed different results than those decorated at HVR1. Transgene product expression from Ad HVR5 was slightly reduced, while transgene product expression from Ad HVR5–αGal was completely abolished, even in a mouse model that does not produce anti-Gal antibodies (C57BL/6N mice). These data were verified by an additional method (quantitative PCR, Fig 8c) and in an additional mouse strain that does not produce anti-Gal antibodies (BALB/c mice). We considered it negligible to analyze *in vivo* transduction by undecorated Ad HVR1 and Ad Fiber, as all analyzed vector constructs did not show differences in *in vitro* transduction efficiency, and comparison of *in vivo* transduction by Ad control and thiol-reactive undecorated Ad HVR5 control (Fig 8a) did not reveal relevant differences. As our *in vitro* analyses did not suggest relevant differences between the distinct vectors, we suggest that there were no differences in the levels of ovalbumin expression *in vivo*.

## Discussion

In the present work, we analyzed the utility of the carbohydrate epitope αGal to induce improved transgene product-directed immune responses by use of *E1*-deleted, replication-defective Ad vectors. While several publications in the past had demonstrated that substantial improvements on the induction of immune responses could be mediated by αGal-decoration of recombinant or attenuated *s*.*c*. vaccines [14–18], importantly, the effects of the trisaccharide αGal on the performance of genetic vaccines remained elusive.

We hypothesized that formation of immunocomplexes composed of αGal-decorated Ad vectors and anti-Gal antibodies, which are naturally present in humans and can be induced in homozygous α-1,3GT KO mice, might lead to an improved uptake of Ad vectors and subsequent transduction of antigen-presenting cells, and an improved generation of CD8 T cells that are reactive to vector-encoded antigens. We utilized a system that allowed for defined chemical glycosylation (αGal-decoration) of Ad vectors at specific capsid sites, and compared the performance of αGal-decorated vectors with their undecorated counterparts when administered via distinct routes.

However, our data revealed that covalent αGal-decoration at the capsid sites hexon HVR5, fiber knob or penton base significantly reduced transgene product-specific CD8 T cell responses induced by immunocomplexed vectors in anti-Gal mice. This suggested that immunocomplexes that had been formed by binding of anti-Gal antibodies to αGal moieties at the capsomers hexon (HVR5), fiber or penton might have been phagocytosed *in vivo* without substantial transduction of cells. Interestingly, immunocomplexes formed after modification of hexon HVR1 maintained their potency to induce transgene product-specific CD8 T cell responses, and thus must have maintained their ability to transduce cells. This finding is of particular importance, since it strongly suggests that in humans with natural pre-existing immunity to Ad, the fate of Ad immunocomplexes may depend on the location of the epitope that is recognized by pre-existing antibodies. In fact, it has been discussed if the fate of Ad immunocomplexes in anti-Ad preimmune individuals has contributed to the puzzling results of the STEP trial. [51,52]

Analysis of the vector-directed CD8 T cell responses (Fig 7) revealed that αGal-decoration of Ad—independent of position and copy number—mildly dampened these responses. This was in agreement with a slight yet statistically significant reduction of anti-Ad IgG titers induced by αGal-decorated vectors compared to undecorated controls (data not shown).

Finally, to determine the ability of the different Ad immunocomplexes to transduce cells *in vivo*, we intravenously injected Ad vectors being αGal-decorated at hexon (HVR1 or HVR5) or fiber, and determined the extent of liver transduction 72 h post injection. While vectors with undecorated capsids exhibited strong liver transduction, αGal-decoration of fiber led to a slight reduction of liver transduction. Importantly, vectors being αGal-decorated at hexon HVR1 or HVR5 barely transduced liver. Since immunocomplexes formed by vectors decorated with αGal at hexon HVR1 were still able to induce OVA-specific CD8 T cell responses and thus able to transduce cells, we speculate that the size of the immunocomplexes directed to hexon HVR5 might have been too large to allow access to hepatocytes. Overall, our data demonstrate that decoration of Ad-based vectors with αGal did not improve their ability to induce transgene product-directed CD8 T-cell responses.

Previously, αGal/anti-αGal immunocomplexation had been described as a functional adjuvant system in anti-Gal mice. Improved cellular immunity had been observed after *s*.*c*. delivery of distinct αGal-decorated antigens. [14–18] While successful for pure antigens, the concept of αGal-decoration had not been tested with *de novo* antigen-expressing vectored vaccines, such as Δ*E1* Ad. However, we did not detect increased immune responses via none of the immunization routes (*i*.*m*., *i*.*p*., *s*.*c*.) following single- or multi-dose immunization. Immune responses were neither improved on the CD8 T cellular nor on the humoral level, and regarding immune responses directed to the vector-expressed transgenic neoantigen, an exemplary vector-expressed Ad antigen, the Ad capsid or the αGal moiety. One potential explanation for this discrepancy might be that we used a relatively low copy number of αGal epitopes per particle. However, the *in vivo* data on liver transduction strongly suggested that Ad immunocomplexes were formed after injection of the vectors. Nevertheless, alternative chemical or biological approaches that enable the generation of more densely αGal-decorated vectors may be required to answer this question. As a further difference, while we applied only αGal-decorated adenoviral vectored vaccines, αGal-decorated recombinant or inactivated protein vaccines (*s*.*c*.) contained Ribi adjuvant which has been reported to promote Th1-type responses. [53,54]

Our studies did not confirm an immunoenhancing adjuvant effect of Ad–αGal/anti-Gal immunocomplexation similar to that described for αGal-decorated protein antigens. Cellular and humoral vector- and transgenic antigen-directed responses were decreased as a consequence of αGal-decoration and immunocomplexation (AICs) of adenoviral vectored vaccines (Ad–αGal) in anti-Gal mice. While not suitable to improve the performance of Ad vectors as a genetic vaccine, we think that the system presented here can become an important tool for the analysis of Ad immunocomplexes *in vivo* that mimick the human situation to a large degree. Pre-existing antibodies can be re-directed to specific capsid sites and this simulates pre-existing immunity. In future, αGal-decoration of viral capsomers can gain biomedical significance as it can be employed as a universal tool for virological *in vivo* characterization of the formation, biodistribution and neutralization of virus immunocomplexes.
